# “Live and Let Die”: What We Learned From US Healthcare and What Seems to Be Valid in the COVID-19 Pandemic

**DOI:** 10.3389/fmed.2021.633222

**Published:** 2021-06-02

**Authors:** Andreas Gerd Jüttemann, Mathias Wirth

**Affiliations:** ^1^Charité University Hospital Berlin, Institut für Geschichte der Medizin, Berlin, Germany; ^2^Brandenburg Medical School Theodor Fontane, Institut für Anatomie, Neuruppin, Germany; ^3^University of Bern, Institut für Systematische Theologie, Bern, Switzerland

**Keywords:** COVID-19, ethics, ICU, Georgetown, trump administration

In 2020, the USA experienced an extreme situation in which medical professionals had to answer essential medical ethics questions regarding rationing and prioritization. In Germany and Switzerland, the vast majority of people, with or without work, are covered by health insurance, especially in the less affluent classes. Scenes like those in New York toward the end of April, where seriously ill people were being turned away from hospitals, were completely unimaginable. European medicine has learnt a great deal from its US counterpart: both the modern US hospital system and the basic assumptions of US medical ethics have been copied on the other side of the Atlantic. The pandemic shows that US medicine not only has to suspend ethical principles due to the particular situation (e.g., triage) but that some ethical principles can hardly be observed in other situations, as the healthcare system hardly allows this.

During one of its peaks in the ongoing COVID-19 pandemic, for instance in New York City, the wide gulf between the ideals of US medical ethics and the reality of the enormous disparities in the US health system became obvious to a worldwide audience. This has been the case for not only medical ethics but also the US hospital system, which for decades has set the tone worldwide. What do US medical ethics and US hospital construction concretely offer that would help in the context of this pandemic and of a wider future in the sense of enfolding something that is already a prominent part of the US health system? When US President Donald Trump visited a mask factory in Arizona on May 5, 2020, the public relations department selected the song “Live and Let Die” by the band Guns N' Roses as the background music (1). It does not refer to the ethical measures of rationing and prioritization that are currently prevalent in the US healthcare system when we look at the global significance of American medical ethics and the US hospital system in recent decades.

In 2020, most parts of the world experienced an extreme situation in which medical professionals had to answer essential medical ethics questions regarding rationing and prioritization. Some countries, such as Germany, Switzerland, and the USA, had a medically favorable starting position when the pandemic began. Medical supplies and hospital equipment were in such good order that rationing and prioritization in connection with the pandemic has so far only occurred in rare cases (2). However, there was a considerable difference between those countries. Meanwhile we heard about a lot of patients who could not be treated due to a lack of health insurance coverage (3). At present, it is precisely the southern states that are affected, where many people do not have insurance (4, 5). Elsewhere, this situation is impossible: for instance, in Germany and Switzerland, the vast majority of people, with or without work, are covered by health insurance, especially in the less affluent classes. Scenes like those in New York toward the end of April, where seriously ill people were being turned away from hospitals, were completely unimaginable. It was incomprehensible to think that this could happen in the USA, a country that has always set an example to those of us who work, research, and teach as medical ethicists and historians (6). But it should come as no surprise, because the reality is this problem has always existed in the US healthcare system, but now it has become more apparent in the context of the pandemic. The contradiction with medical ethics is also more apparent here, as it has now become clear to the entire world for the first time.

More than 40 years ago, two US medical ethicists, Beauchamp and Childress (7), first published their four principles under the title *Principles of Biomedical Ethics*. Both probably taught the four basic assumptions to their students in such a prayer-mill-like canon that they called it the “Georgetown Mantra”: respect for *autonomy, non-maleficence, beneficence*, and *justice*. The Georgetown Mantra became the most influential text for the development of the discipline of medical ethics, not only in the USA, but also around the world. In recent years, research in ethics has been searching for answers to this question of humane healthcare with the help of the Georgetown Mantra: can the patient maintain self-determination in the absence of health insurance coverage, especially in times of a pandemic? How do I come to a decision in a triage situation? Do I fulfill my duty of care as a hospital employee? Do I not harm the patient? Is there social justice in answering a medical–ethical dilemma? In most university hospitals, this mantra is an integral part of medical education. However, also many current examples from the USA show medical ethicists from Europe that US medicine in particular is finding it difficult at present to recognize two of the most widely replicated principles developed at Georgetown University. In the following, we would like to explain some current examples based on the mantra:

- *Beneficence*: At the beginning of the 20th century, medical awareness in the USA grew for a holistic treatment of diseases and especially for a functional optimization of inpatient facilities for diagnostics and care (8). This required adequate structures within the hospital. Closer cooperation of physicians within one hospital was the only logical solution. In Rochester (Minnesota), William Worrall Mayo was the first to establish a large community hospital where numerous specialists could work together under one roof. Doctors should become team workers and no longer be individual rulers in their specialist clinics. The new Mayo Clinic, opened in 1914, received worldwide attention (9). Several architects oriented themselves to the coordinated diagnostic and therapeutic processes in this clinic. The Mayo Clinic not only revolutionized the US hospital system, but it also became an export hit.
  The system was adopted in Europe, even though it took time to be established: in 1958, the US architect Arthur Q. Davis was only able to achieve the first prototypical (public) hospital building in accordance with the Mayo system in West Berlin thanks to the idealistic and financial support of the US State Department (10). Until the opening of the new Berlin Medical Center 50 years ago in 1969, German chief physicians had never known “teamwork” carried out on such a scale (10). And again, a US model of *beneficence* was adapted in Europe and began its triumphal march.
- *Justice:* The question of social justice, which in the Georgetown Mantra is actually the central fourth element of any basic medical–ethical consideration, (11) had to be completely ignored, particularly in the USA: Beauchamp and Childress' principle demands fair distribution of healthcare administration, capacities, and resources; that is, equal cases should be treated equally. Some COVID-19 patients in the USA often have poor or no health insurance coverage, and a large proportion of them belong to a less affluent class (4, 5). This is despite the fact that intensive care unit (ICU) bed capacity in the USA at 29.7 (12) [like Germany's at 38.7 (13)] is one of the highest per-capita numbers in the world, far more than many other European countries (Sweden at 5.8 and the UK at 6.6) (14).
  In Sweden, in principle, every resident is covered by state health insurance through income tax; even people who do not pay taxes, however, are entitled to medical care (15). In the UK, everyone who is a resident is automatically insured through the National Health Service (NHS). There are no health insurance contributions there. Everyone who does not work is also insured as long as they are registered as a resident. Public healthcare for the entire population is financed by taxpayers' money (doctors and nurses are directly employed by the NHS), so that almost all medical services are free of charge for all residents (except drugs and dental services) (16).
  In Germany, every employer must pay regular contributions to health insurance companies based on income (there are standard legal insurance and private insurance with better benefits for self-employed or people with higher income). These health insurances then reimburse services that are insured in the event of illness. Unemployed persons are insured through the employment office. There is an insurance obligation. Uninsured people exist but are fairly rare (17).
- *Non-maleficence*: When US President Trump visited that mask factory in Arizona, “Live and Let Die,” which many found cynical in view of the simultaneous situation in many US states, can also be seen as a metaphor for a current medical–ethical question. For years, in almost all countries of the world, medical ethics has been discussing the questions of rationing and prioritization in the face of scarcity of funds in the healthcare system (18).
- *Autonomy*: There would certainly be other examples to show that basic principles of medical ethics according to Beauchamp and Childress cannot be fulfilled in the USA at present: the fact that ventilation was initially applied in such a way that no critical perspective of the negative consequences was considered contradicts this approach. There might also be COVID-19 patients who prefer palliative therapy.

European medicine has learnt a great deal from its US counterpart: both the modern US hospital system and the basic assumptions of US medical ethics have been copied on the other side of the Atlantic. Both Beauchamp and Childress as well as Franklin, Mayo, and Davis were not only pioneers for the healthcare system in the USA but have had far-reaching significance in the history of hospitals and ethics of medicine in Europe to this day.

But at this point, an appeal should be made again based on the ongoing experience of the COVID-19 pandemic for a fair hospital system closely following Franklin's and Mayo's models and the ideas in the Georgetown Mantra.

The normal ethical principles are replaced by triage. The pandemic shows that US medicine not only has to suspend ethical principles due to the particular situation (e.g., triage) but that some ethical principles can hardly be observed in other situations, as the healthcare system hardly allows this. With this text, we want to launch a discussion as to why so many achievements, which were once established by US university ethics and which are taught worldwide in order to make even better use of the achievements of the US hospital system, have fallen into oblivion in the USA today.

## Author Contributions

AJ focused on the history and hospital system and MW on the ethical assessment.

## Conflict of Interest

The authors declare that the research was conducted in the absence of any commercial or financial relationships that could be construed as a potential conflict of interest.
